# Intra-Articular Injection of (-)-Epigallocatechin 3-Gallate to Attenuate Articular Cartilage Degeneration by Enhancing Autophagy in a Post-Traumatic Osteoarthritis Rat Model

**DOI:** 10.3390/antiox10010008

**Published:** 2020-12-23

**Authors:** Hsuan-Ti Huang, Tsung-Lin Cheng, Cheng-Jung Ho, Han Hsiang Huang, Cheng-Chang Lu, Shu-Chun Chuang, Jhong-You Li, Tien-Ching Lee, Shih-Tse Chen, Yi-Shan Lin, Chih-Yao Lee, Lin Kang, Sung-Yen Lin, Chung-Hwan Chen

**Affiliations:** 1 Orthopaedic Research Center, Kaohsiung Medical University, Kaohsiung 80701, Taiwan; hthuang@kmu.edu.tw (H.-T.H.); junglecc@kmu.edu.tw (T.-L.C.); u105801010@kmu.edu.tw (C.-J.H.); cclu@kmu.edu.tw (C.-C.L.); f86225016@ntu.edu.tw (S.-C.C.); u109508016@kmu.edu.tw (J.-Y.L.); u103800014@kmu.edu.tw (T.-C.L.); r950084@kmu.edu.tw (Y.-S.L.); u103000041@kmu.edu.tw (C.-Y.L.); 2 Department of Orthopedics, Kaohsiung Medical University Hospital, College of Medicine, Kaohsiung Medical University, Kaohsiung 80701, Taiwan; 3 Regeneration Medicine and Cell Therapy Research Center, Kaohsiung Medical University, Kaohsiung 80701, Taiwan; 4 Departments of Orthopedics, College of Medicine, Kaohsiung Medical University, Kaohsiung 80701, Taiwan; 5 Department of Orthopedics, Kaohsiung Municipal Ta-Tung Hospital, Kaohsiung 80145, Taiwan; 6 Department of Physiology, College of Medicine, Kaohsiung Medical University, Kaohsiung 80701, Taiwan; 7 Graduate Institute of Medicine, College of Medicine, Kaohsiung Medical University, Kaohsiung 80701, Taiwan; 8 Department of Veterinary Medicine, National Chiayi University, Chiayi 60004, Taiwan; hhuang@mail.ncyu.edu.tw; 9 Department of Orthopedics, Kaohsiung Municipal Hsiao-Kang Hospital, Kaohsiung Medical University, Kaohsiung 80701, Taiwan; 10 Department of Psychiatry, National Taiwan University Hospital Hsin-Chu Branch, Hsin Chu 30059, Taiwan; chenshihtse108@gmail.com; 11 Department of Nursing, Yuanpei University of Medical Technology, Hsinchu 30015, Taiwan; 12 Department of Obstetrics and Gynecology, National Cheng Kung University Hospital, College of Medicine, National Cheng Kung University, Tainan 70101, Taiwan; 13 Department of Healthcare Administration and Medical Informatics, Kaohsiung Medical University, Kaohsiung 80701, Taiwan; 14 Institute of Medical Science and Technology, National Sun Yat-Sen University, Kaohsiung 80424, Taiwan

**Keywords:** (-)-epigallocatechin 3-gallate (EGCG), apoptosis, autophagy, cartilage, mTOR, post-traumatic osteoarthritis

## Abstract

(-)-Epigallocatechin 3-gallate (EGCG) is the main active green tea catechin and has a wide variety of benefits for health. Post-traumatic osteoarthritis (PTOA) occurs as a consequence of joint injuries that commonly happen in the young population. In this study, we investigated the effects of EGCG on PTOA prevention by using the anterior cruciate ligament transection (ACLT)–OA model and further investigated the roles of autophagy in OA treatment. Our results showed that intra-articular injection of EGCG significantly improved the functional performances and decreased cartilage degradation. EGCG treatment attenuated the inflammation on synovial tissue and cartilage through less immunostained cyclooxygenase-2 and matrix metalloproteinase-13. We further noted EGCG may modulate the chondrocyte apoptosis by activation of the cytoprotective autophagy through reducing the expression of the mTOR and enhancing the expression of microtubule-associated protein light chain 3, beclin-1, and p62. In conclusion, intra-articular injection of EGCG after ACL injury inhibited the joint inflammation and cartilage degradation, thereby increasing joint function. EGCG treatment also reduced the chondrocyte apoptosis, possibly by activating autophagy. These findings suggested that EGCG may be a potential disease-modifying drug for preventing OA progression.

## 1. Introduction

Post-traumatic osteoarthritis (PTOA) frequently occurs as a sequela of joint injury. Intra-articular incongruity or joint instability after acute joint injury increases the risk of PTOA. Despite the advances in surgical techniques and the novel developments of instruments, joint reconstruction surgery has not been shown to reduce the rate of PTOA [1,2,3,4]. Chondrocytes are the only resident cells responsible for maintaining the hemostasis of articular cartilage. The chondrocyte death and extracellular matrix (ECM) breakage caused by a high-energy joint injury triggers a degenerative cascade and has a significant role in the development of PTOA [5,6]. Chondrocyte apoptosis is the central feature in the early pathogenesis of PTOA, and prevention of chondrocyte apoptosis can reduce the severity of OA [7,8,9,10]. Due to the limited regenerative capacity of chondrocytes, the prevention of chondrocyte death after injury is mandatory for the prevention of PTOA.

(-)-Epigallocatechin 3-gallate (EGCG), the most plentiful bioactive polyphenol of green tea, is a potential anti-arthritic agent because of its anti-inflammatory effect and anti-oxidant effect [11,12]. Studies have shown that EGCG inhibits nitric oxide, cyclooxygenase-2 (COX-2), and the production of prostaglandin E2 in human OA chondrocytes stimulated with interleukin-1β (IL-1β) [13,14]. EGCG has also been reported to be able to inhibit the expression of pro-inflammatory genes (i.e., COX-2, tumor necrosis factor-α (TNF-α), matrix metalloproteinases (MMP)-1, MMP-13, iNOS, and a disintegrin and metalloproteinase with thrombospondin motifs (ADAMTS)-5). In addition to anti-inflammation effects, EGCG can further reduce the ECM degradation by inhibition of the activity of MMPs ADAMTS. EGCG can suppress the advanced glycation end products stimulated by the catabolic response in human OA chondrocytes by inhibiting the activity of TNF-α and MMP-13 [15]. It has been also reported that EGCG can dose-dependently inhibit the activity of MMP-1 and MMP-13 in human cartilage explants stimulated with IL-1β [16]. Moreover, EGCG can also reduce cartilage aggrecan breakdown by selectively suppressing the activity of ADAMTS-1, 4, and 5 [17]. These studies supported the chondroprotective effects of EGCG and further indicated that EGCG may be a potent disease-modified agent in OA.

Autophagy is a highly regulated self-renewing process that can selectively remove the damaged organelles or misfolded proteins, subsequently increasing the threshold of the stress required to induce apoptosis [18]. Emerging evidence has shown that autophagy reduction is closely related to aging-related disease, and the induction of autophagy prolongs cellular longevity and promotes its function [19]. OA is a typical aging-related joint disease and is also associated with autophagy dysfunction. Caramés et al., demonstrated that human OA, age, and surgically-induced OA in mice were associated with reduced expression of autophagy markers and an increase of apoptosis [20]. The mammalian target of rapamycin (mTOR) inhibitor, rapamycin, can promote autophagy function and decrease cell death and matrix damage [21]. Autophagy dysfunction is closely related to the pathogenies of OA, and enhancing autophagy in chondrocytes may serve as an effective treatment strategy for OA.

The majority of the literature has established the benefit of EGCG for inhibition of OA progression, mainly by the anti-inflammatory effect. The role of autophagy in OA cartilage in response to EGCG remained unknown until our research. The present study aimed to examine the effect of intra-articular injection of EGCG on articular cartilage in an anterior cruciate ligament transection (ACLT) induced OA model. Moreover, the effects of EGCG on the activity of autophagy in OA cartilage were investigated as well. We hypothesized that EGCG can reduce cartilage degradation in experimental OA animals by upregulating autophagy function.

## 2. Materials and Methods

### 2.1. Experimental Animals and Animal Model

All experimental protocols were conducted according to the “Guide for the Care and Use of Laboratory Animals” of Kaohsiung Medical University and with the approval of the Kaohsiung Medical University Institutional Animal Care and Use Committee (IACUC approval number 103160). Thirty-six 11-wk-old male Sprague–Dawley rats with no radiographic evidence of joint disease were used for the study. The experimental animals were randomly allocated to three groups by body weight and the results of the endurance test as follows: the control group comprised 12 rats that underwent sham surgery and received vehicle treatment; the anterior cruciate ligament transection (ACLT) [22,23] group comprised 12 rats that underwent ACLT surgery and also received vehicle treatment; and the OA + EGCG group comprised 12 rats that underwent ACLT surgery and received 10 μM EGCG treatment. The mean ± SD weight of the rats was 357.8 ± 53.7 g.

Twenty-four rats underwent unilateral (right knee joint) ACLT via medial parapatellar arthrotomy. The knee joint was exposed after the lateral subluxation of the patella. The ACL was transected near the tibial insertion under direct vision. The anterior draw test was performed to confirm the complete transection of ACL. The operated leg was not immobilized. Rats were allowed free activity in their cages. The blood samples were collected to determine serum glutamate-pyruvate transaminase (SGPT), serum glutamate-oxaloacetate transaminase (SGOT), blood urea nitrogen (BUN), creatinine (CRE), and high sensitivity C-reactive protein (hsCRP) levels after the full course of EGCG treatment. Control and experimental rats were euthanized at 18 weeks old by CO_2_ gas inhalation after the weight-bearing distribution test and treadmill exercise assessment.

### 2.2. Drug Treatment

EGCG (E4143) was purchased from Sigma-Aldrich (St. Louis, MO, USA) [24,25,26,27,28,29,30]. Purity was 99.6% at room temperature. The rats received treatments with 40 μL of EGCG at the concentration of 10 μM or the same volume of phosphate-buffered saline (PBS) according to the experimental designs. EGCG or vehicle was applied by intra-articular injection once every three days for 5 weeks, 2 weeks after the operation.

### 2.3. Weight-Bearing Distribution Test

The weight distribution of each hind paw was measured using a dual-channel weight averager (Singa Technology Corporation, Taipei, Taiwan). The difference of hind paw weight bearing between the studied (right) and the normal (left) limb was determined as an index of joint discomfort in the OA knee. The assessments were regularly performed on weekly intervals 1 week before ACLT surgery until the rats were euthanized. Briefly, a rat was placed in an angled acrylic chamber and positioned so that each hind paw rested on a separate force plate. The force exerted by each hind limb was averaged over a 5 s period, and each data point was the mean of three 5 s readings [22].

### 2.4. Endurance Test

Animals were acclimated to the test after a training program that consisted of a running exercise on Columbus instruments rodent treadmill (Columbus, OH, USA) at a speed of 10–15 m/min, with 15 min/day for 1 week before ACLT surgery. Measurement was performed three times in each rat, and the average data were calculated. The treadmill was set at a velocity of 35 m/min. The test was terminated at the maximum time of running endurance [22,23,31].

### 2.5. Histological Analysis

For histological analysis, the specimens were fixated in 10% buffered formalin for 24 h, followed by decalcified in 10% formic acid. Samples were embedded in paraffin blocks and prepared in the coronary plane with 5 μm thickness for staining. Glycosaminoglycan (GAG) was stained with safranin O-fast green (1% safranin O counterstained with 0.75% hematoxylin and then 1% Fast Green; Sigma) and quantified with Image-Pro Plus 5.0 software (Media Cybernetics, Rockville, MD, USA). The relative density of the red-stained area to the total area (density/total area) in each group was calculated [22,32]. The progression of OA was assessed using a microscopic scoring system according to the Osteoarthritis Research Society International’s (OARSI) guidelines [33]. The synovial inflammation was examined using a histopathological grading system for chronic synovitis [34]. The degree of synovium inflammation can be measured by scoring the histological findings of three parameters, including the hyperplasia/enlargement of the synovial lining cell layer, activation of resident cells/synovial stroma, and inflammatory cell infiltration.

### 2.6. Immunohistochemically Staining (IHC) for Type II Collagen (Col II), Type X Collagen (Col X), Cyclooxygenase-2 (COX-2), Matrix Metallopeptidase 13 (MMP-13), and Markers of Autophagy

Paraffin-embedded slides were deparaffinized in xylene and rehydrated in grade ethanol. The samples were blocked with 3% hydrogen peroxide and then blocked with fetal bovine serum for 1 h. The slides were then incubated using the monoclonal antibody to Col II (Chemicon International, Temecula, CA, USA), Col X (COSMO, Tokyo, Japan), COX-2 (sc-7951,Santa Cruz, CA, USA), MMP-13 (ab39012, Abcam, Cambridge, MA, USA) [35], mTOR (EnoGene, New York, NY, USA), microtubule-associated protein light chain 3 (LC3) (14600-1-AP, Proteintech, Rosemon, IL, USA), beclin 1 (11306-1-AP, Proteintech, Rosemon, IL, USA), and p62 (GeneTex, Irvine, CA, USA) at 37 °C for 4 h. After incubation with a primary antibody, an EXPOSE mouse and rabbit-specific horseradish peroxidase-diaminobenzidine detection IHC kit (Abcam, Cambridge, MA, USA) was applied. Finally, sections were counterstained with hematoxylin. The data were quantified using Image-Pro Plus, by defining the immunostaining of positive cells normalized with total cells.

### 2.7. Terminal Deoxynucleotidyl Transferase dUTP Nick end Labeling Stain (TUNEL Staining)

We used TUNEL staining (In Situ Cell Death Detection Kit, POD, Roche Applied Science, Germany) to detect the DNA fragments in an apoptotic cell. The procedures were performed according to the manufacturer’s protocol. DAPI-stained cells were counted in the center area of the cartilage in the tibial plateau. The apoptotic rate of chondrocytes was defined as the ratio of red-stained cells (dead cells) to blue-stained cells (total cells) [36].

### 2.8. Statistical Analysis

All data are presented as means ± SEs. Comparisons of the data were analyzed using one-way ANOVA, and multiple comparisons were conducted by Scheffé’s post hoc test using SPSS (version 17.1 for Windows; SPSS, Chicago, IL, USA). *p* <  0.05 was considered statistically significant.

## 3. Results

### 3.1. Intra-Articular Injection of EGCG Does Not Interfere with Hepatic Function, Renal Function, or Inflammation

Concerned with the possible hepatic and renal toxicity of EGCG, we collected the blood samples to monitor the liver enzyme, renal function, and inflammation markers. There were no significant differences in SGOT, SGPT, BUN, CRE, and hsCRP between the OA and the EGCG-treated groups. Moreover, the multiple joint injections (one injection per 3 days for 5 weeks) did not induce joint inflammation in each group (Table 1).

### 3.2. Intra-Articular Injection of EGCG Attenuates OA Symptoms and Improves Running Endurance

**Figure 1:**
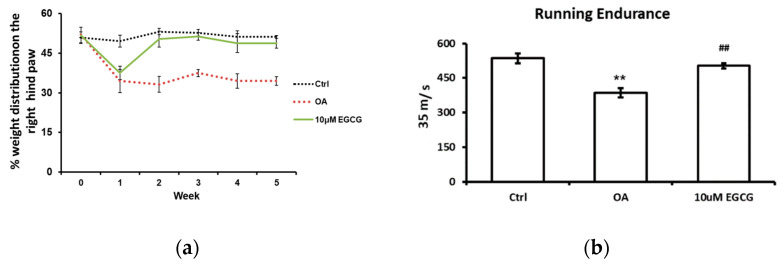
The anti-allodynic effect of EGCG. (**a**) The temporal pattern of weight distribution of the osteoarthritis (OA) joint. Rats injected with 10 μM EGCG (EGCG group) or saline (OA group and control group) in the right knee were examined for the change in hind paw weight distribution for 5 weeks after anterior cruciate ligament transection (ACLT). A significant difference was noted between OA and EGCG-treated groups at 2, 3, 4, and 5 weeks after ACLT. (**b**) The results of running endurance at 5 weeks after ACLT. The running endurance was tested one week before and every one week after ACLT for 5 weeks. The running endurance was significantly decreased in the OA group compared with the control. There was a significant improvement in running endurance after EGCG treatment. (** *p* < 0.01 OA vs. control; ## *p* < 0.01 OA vs. EGCG).

The weight-bearing ability of the OA limb and the running endurance were tested to investigate whether EGCG attenuates OA symptoms and improves the functional performance after ACLT. The weight-bearing distribution on the right hind paw during the experimental period is shown in Figure 1a. During the experiments, there was no obvious change in the weight-bearing distribution pattern in the control group. We observed a significant decrease in the weight-bearing ability on the right hind paw at each checkpoint after ACLT (*p* < 0.01 compared with the control group). In contrast, for the EGCG-treated group, the values reduced in the first week, but significantly improved in the second week and thereafter until the end of the study (*p* < 0.01 compared with OA group).

The treadmill test was used as an endurance assessment, and the results are shown in Figure 1b. The rats in the control group could endure 535 s of running, whereas the rats in the OA group could only endure 386 s (*p* < 0.01). Intra-articular injection of EGCG improved the running endurance of rats after ACLT. The running endurance of EGCG-treated rats was restored to 503 s; that is a significant improvement compared with ACLT rats (*p* < 0.01).

### 3.3. Intra-Articular EGCG Injection Delays the Cartilage Degradation

**Figure 2:**
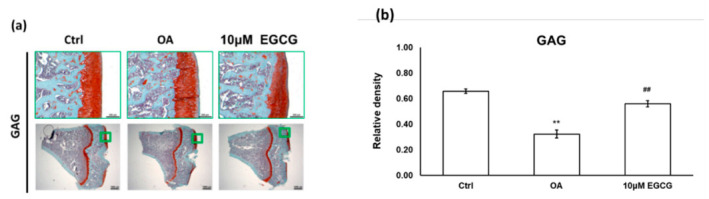
The histological analysis of osteoarthritis (OA) and the quantitative assessment of glycosaminoglycan (GAG) loss in the anterior cruciate ligament transection (ACLT) rats. (**a**) The representative micrographs of the proximal tibial cartilage with safranin O-fast green staining. (**b**) The quantitative analysis of GAG loss. No obvious arthritic changes or GAG loss was observed in the control group, but marked GAG loss was observed in the knee joints of ACLT rats. Meanwhile, the GAG loss was reduced after EGCG treatment. (** *p* < 0.01 OA vs. control; ## *p* < 0.01 OA vs. EGCG).

We used a joint instability-induced OA model to investigate the role of EGCG in the development of OA. We observed obvious cartilage damage to the articular surface and marked GAG loss in the ACLT rats that demonstrated successful OA induction. The relative density of stained GAG in the OA group was significantly lower than that in the control group (*p* < 0.01). Meanwhile, we also found that intra-articular EGCG injection decreased the severity of OA, including presenting less cartilage erosion and GAG loss compared with OA rats (Figure 2a). As shown in Figure 2b, the results of the quantification analysis showed that the relative density of stained GAG in the EGCG-treated group was significantly higher than that of the OA group (*p* < 0.01) (Figure 2).

These results were further confirmed by the OARSI score, which is commonly used to evaluate the histopathological presentation of cartilage degeneration. In the OA group, the averaged OARSI score was 5.2 ± 0.5, which was significantly higher than that in the control group (2.6 ± 0.2). Intra-articular EGCG injection alleviated the cartilage degradation by presenting markedly lower OARSI scores (3.0 ± 0.29) compared with ACLT rats (Table 2).

### 3.4. Intra-Articular Injection of EGCG Reduces the ECM Degradation

**Figure 3:**
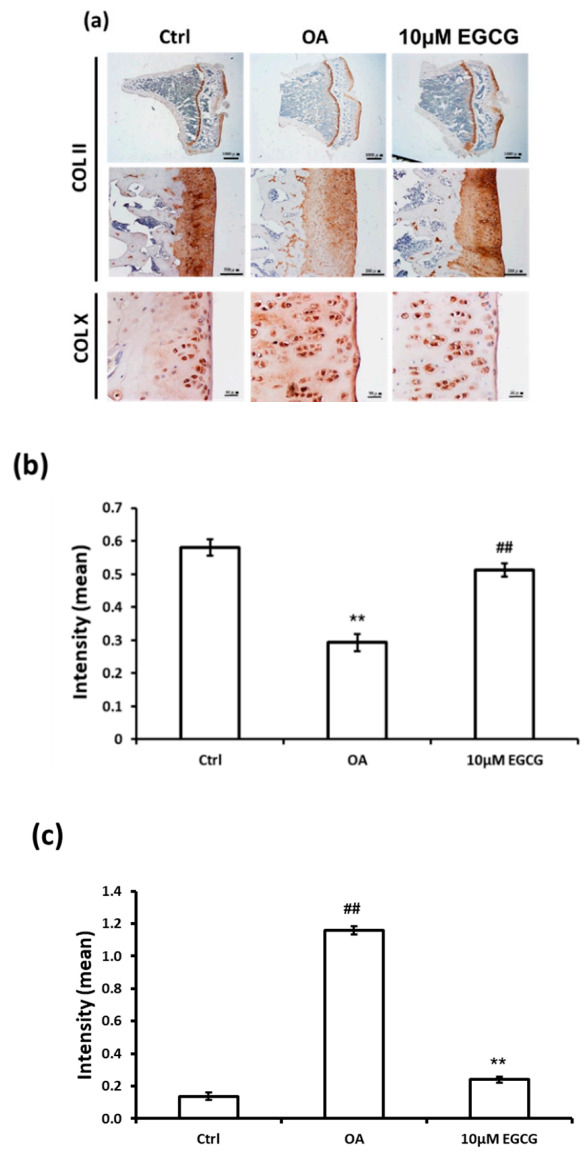
Immunohistochemistry (IHC) of collagen type II (Col II) and collagen type X (Col X) in the articular cartilage of control, osteoarthritis (OA), and OA+EGCG joints. (**a**) The representative micrographs of IHC analysis for Col II and Col X. (**b**) The quantitative analysis of immunostained Col II. (**c**) The quantitative analysis of immunostained Col X. The immunostained Col II was reduced and the immunostained Col X was enhanced after anterior cruciate ligament transection (ACLT). There was significantly more staining of Col II and less staining of Col X in the EGCG-treated rats compared with ACLT rats. (** *p* < 0.01 OA vs. control; ## *p* < 0.01 OA vs. EGCG).

The IHC staining was used to investigate whether intra-articular injection of EGCG ameliorates the OA progression by decreasing the ECM degradation. The representative micrographs of Col II and Col X IHC staining are illustrated in Figure 3a. The results of quantitative analysis demonstrated that Col II staining was significantly reduced in the OA group compared with the control group (*p* < 0.01). The densities of immunolocalized Col II staining in both control and EGCG-treated groups were higher than those of the OA groups (Figure 3b). In contrast, the density of immunolocalized Col X was strongly expressed in the OA group, but only minimal Col X-stained chondrocytes were found in the control group. Col X was weakly expressed in the cartilage of EGCG-treated rats, but it was more extensively visible in the ACLT rats (Figure 3c).

### 3.5. Intra-Articular Injection of EGCG Decreases Joint Inflammation in ACLT Rats

**Figure 4:**
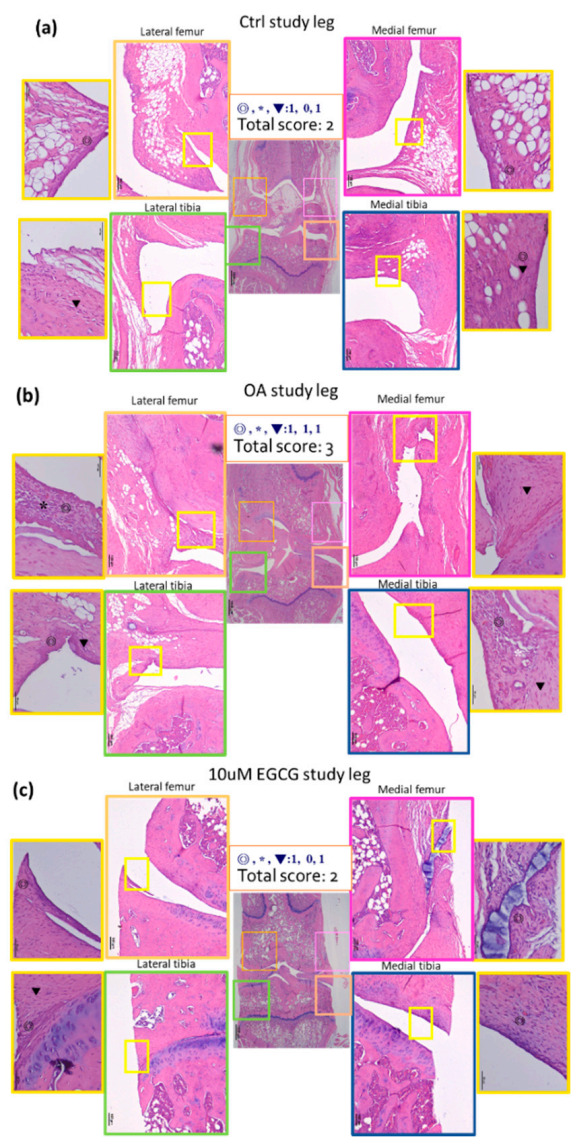
Representative sections of synovial membrane from both knee joints of rats. (**a**) Sample from a rat after sham operation (control group). Note the low-grade inflammatory cell infiltrate and the mild increases of synovial hyperplasia and stroma activation. (**b**) Sample from a rat after anterior cruciate ligament transection (OA group), in which there is more infiltration by inflammatory cells. (**c**) Sample from a rat after ACLT and treated with EGCG (10 μM EGCG group) shows similar sham-operated joints with a fewer infiltrating cells than the OA joint.

Hematoxylin and eosin histology was performed on the synovial tissues from both knees of rats. The representative micrographs of synovial tissues histology are shown in Figure 4. There was almost no inflammation in the contralateral knee and a mild increase of inflammation after a sham operation in the control group. An increase of synovial inflammation was noted in ACLT rats. In order to evaluate the severity of inflammation, we used a histological semi-quantitative scoring system for evaluation of the inflammation in the synovial tissue and the results were demonstrated in Table 3. The synovial tissue from the ACLT rats had significantly higher inflammation scores (3.17 ± 0.41) compared to that of the control group (2.50 ± 0.0) (*p* < 0.01). The inflammatory response of the EGCG-treated group was significantly alleviated compared with that of the OA groups (*p* < 0.05). These results indicated a decrease in synovial inflammation in response to EGCG treatment (Figure 4).

**Figure 5:**
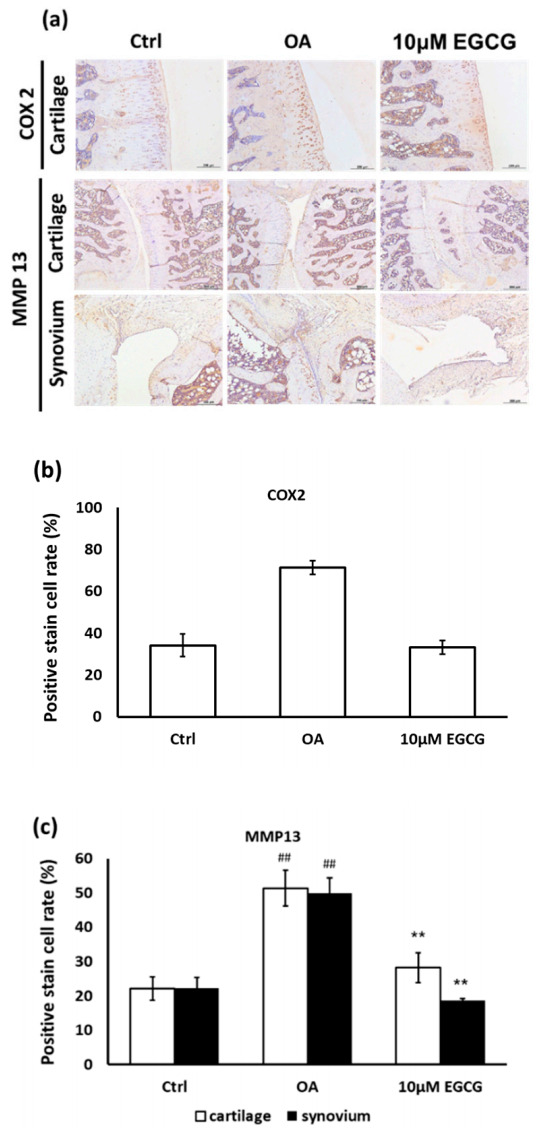
Immunohistochemistry (IHC) of cyclooxygenase-2 (COX-2) and matrix metalloproteinases-13 (MMP-13) in the articular cartilage of control, osteoarthritis (OA), and OA + EGCG joints. (**a**) The representative micrographs of IHC analysis for COX-2 and MMP-13. Magnification: 10×. (**b**) The quantitative analysis of immunostained COX-2. Magnification: 40×. (**c**) The quantitative analysis of immunostained MMP-13 Magnification: 40×. Sections of osteoarthritic cartilage demonstrated more staining of COX-2 and MMP-13 protein compared to samples from control group. In the EGCG-treated joint, the densities of immunostained COX-2 and MMP-13 were significantly reduced. (** *p* < 0.01 OA vs. control; ## *p* < 0.01 OA vs. EGCG).

Moreover, the anti-inflammatory effects of EGCG on OA cartilage were further confirmed by IHC staining. The represented micrographs of immunostained COX-2 and MMP-13 protein are shown in Figure 5a. The results indicated that COX-2 and MMP-13 expression in ACLT rats was significantly increased compared with the control group. The treatment of EGCG in ACLT rats exhibited significantly less inhibited density of immunolocalized COX-2 and MMP-13 proteins compared with ACLT rats (Figure 5b,c).

### 3.6. Intra-Articular Injection of EGCG Prevents Chondrocyte Death

**Figure 6:**
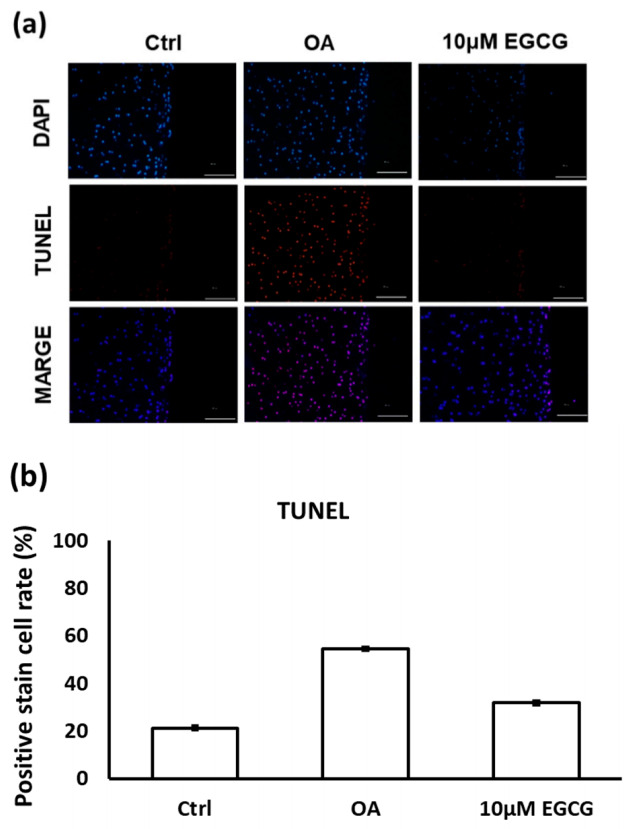
(**a**) The representative micrographs of TUNEL staining showing an increase of TUNEL-positive cells after osteoarthritis (OA) induction. Notably, fewer TUNEL-positive cells were observed in EGCG-treated OA joints. (**b**) Quantitative analysis of chondrocyte apoptosis in three groups. (** *p* < 0.01 OA vs. control; ## *p* < 0.01 OA vs. EGCG).

The results of TUNEL staining indicated that EGCG treatment prevented cell apoptosis in the experimental OA cartilage (Figure 6a). The apoptotic rate of chondrocytes in the cartilage of the OA group rats (54.5 ± 4.9%) was significantly higher than it was in the control group rats (21.2 ± 2.8%). However, as shown in Figure 6b, the TUNEL positively-stained cells were significantly decreased when treated with EGCG (31.8 ± 2.3%).

### 3.7. Intra-Articular Injection of EGCG Increases Autophagy

**Figure 7:**
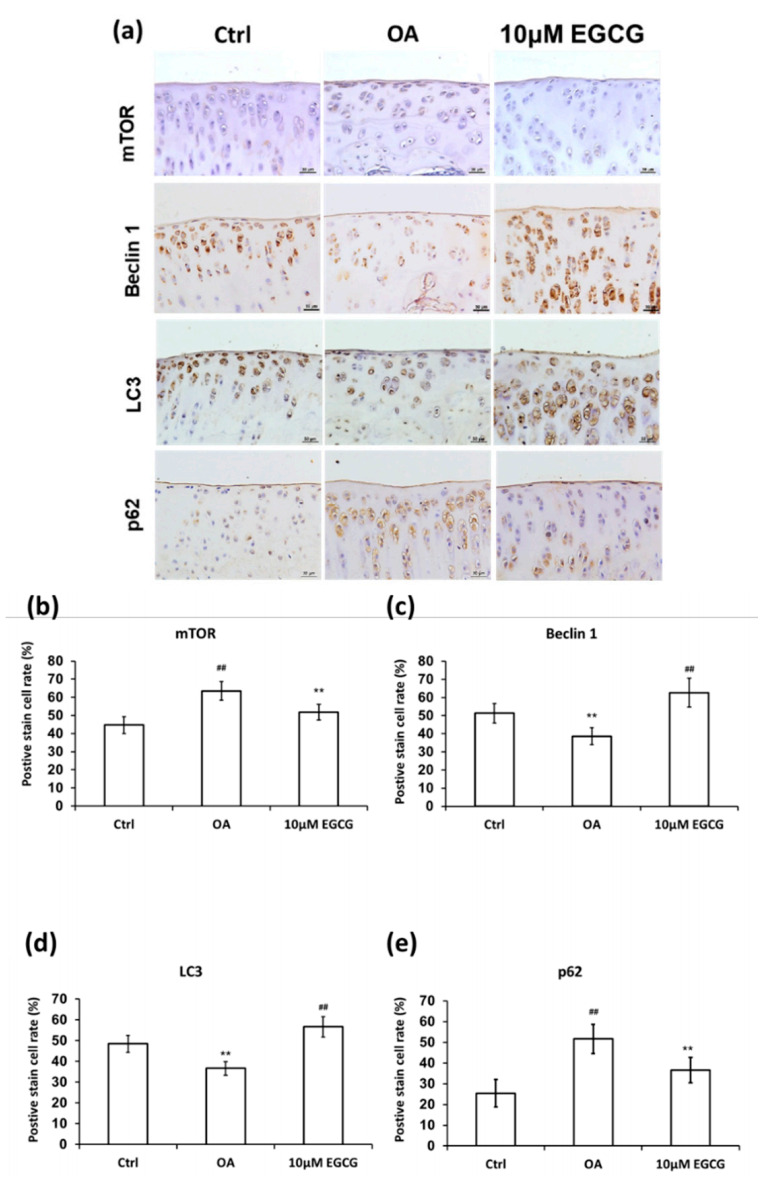
Immunohistochemistry (IHC) of autophagy-related proteins in articular cartilage of control, osteoarthritis (OA), and OA+EGCG joint. (**a**) The representative micrographs of the mechanistic target of rapamycin (mTOR)-, beclin-1-, light chain 3 (LC3)-, and p62-immunostained articular cartilages from the joints of control, osteoarthritis (OA), and OA+EGCG groups. The positive stained cell ratio of autophagy-related proteins were measured and compared among groups. The results of mTOR were shown in (**b**), beclin-1 were shown in (**c**), LC3 were shown in (**d**), and p62 were shown in (**e**) (** *p* < 0.01 OA vs. control; ## *p* < 0.01 OA vs. EGCG).

To clarify whether EGCG treatment can rescue the autophagy effect in the experimental OA model, we examined the expression of autophagy markers, including mTOR, beclin-1, LC3, and p62 in cartilage samples (Figure 7). The analysis of IHC staining showed that autophagy dysfunction in response to OA induction was rescued by EGCG. The expression of mTOR in the EGCG group was significantly lower than that in the OA group in the tibial plateau (*p* < 0.01). Meanwhile, the densities of beclin 1 and LC3 in the cartilage of both control and EGCG-treated group were significantly higher than in the OA group (*p* < 0.01). In contrast, the immunolocalized p62 in the cartilage of both control and EGCG-treated groups was significantly more prevalent than that in the OA group (*p* < 0.01).

## 4. Discussion

OA is the most prevalent joint disease causing a substantial economic burden worldwide. There is no currently available disease modifying agent to delay the course of OA progression. Here, we showed that early intra-articular injection of EGCG can effectively reduce the arthritic changes in ACLT rats not only mediated by reducing the inflammation but also reducing the chondrocyte apoptosis by enhancing autophagy function. Our results showed that EGCG-treated rats exhibited less cartilage erosion and GAG loss, smaller OARSI scores, less synovitis, less MMP-13 and COX-2 (immunolocalized staining), and more autophagy with upregulation of autophagy markers. The dual effect of EGCG through the prevention of inflammation and the promotion of autophagy suggests that EGCG presents as a potential disease-modifying target in OA.

Although the pathology of PTOA is not clear, a robust release of inflammatory mediators in response to joint injury is thought to be a major part of the pathogenesis of PTOA [37]. Inflammatory cytokines including IL-1β, TNFα, and IL-6, and global MMP activity were significantly increased after ACL injury within 24 h [38]. These pro-inflammatory cytokines increase their presence significantly on the first day after injury and can maintain abnormal concentrations in the joint over 6 months after ACL injury [39]. IL-1β and TNF-α, the key pro-inflammatory cytokines of OA, can further stimulate the production of MMPs, causing the degradation of the cartilage matrix, and also promote chondrocytes to secrete cytokines, resulting in more cartilage loss [40]. Early anti-inflammatory intervention is possibly crucial for the prevention of cartilage degradation and further preventing the development of chronic joint pain and disability. EGCG has been shown to have anti-inflammatory effects that can suppress the action of many pro-inflammatory cytokines, such as MMPs and COX-2, by scavenging reactive nitrogen species [41]. The chondroprotective effects of EGCG mediated by reducing inflammation and the catabolic cascade, have also been proven in in vitro studies with human OA chondrocytes [13,42] and in in vivo studies with experimental OA animals [43,44]. Despite having a lesser extent of inflammation than rheumatoid arthritis, synovial thickening and cellular infiltration are not uncommon in PTOA [45]. As in chondrocytes, EGCG can also suppress the IL-1β-induced MMP-2 and TNF-α-induced MMP-1 and 3 production in rheumatoid arthritis synovial fibroblasts [46,47]. In a study using carrageenan induced OA in rats, the green tea extract-treated rats represented a marked reduction of inflammatory cells infiltrating the synovial membrane, and lower levels of lipid peroxides, nitric oxide, and total thiols in plasma [44]. In the present study, we found that EGCG attenuated the inflammation of synovial tissues, decreased the COX-2 and MMP-13 protein expression in cartilage, and further prevented the degradation of Col II. Our findings were consistent with a previous study that intra-peritoneal injection of 25 mg/kg EGCG can decrease the progression of OA and relieve OA-associated symptoms with reduced immunolocalized staining and gene expression of MMP-13 [43]. However, liver damage is a potential concern of EGCG; liver enzyme elevation has been noticed in a clinical study that administered EGCG over 800 mg/day [48]. Unlike the systemic application, the intra-articular application in our study is a safer method for drug administration that only required a relatively smaller dose to achieve a similar chondroprotective effect. A previous study comparing the clinical efficacy of polyarticular corticosteroid injection to systemic administration of on RA demonstrated that the intra-articular injection provided better symptom improvements and significantly fewer systemic adverse effects [49].

Although we did not compare the efficacies of intra-articular injections and systemic applications of EGCG for the prevention of cartilage degradation in this study, we inferred that intra-articular injection would be a more efficacious therapeutic administration for PTOA. Unlike chronic OA, kinds of pro-inflammatory cytokines are significantly elevated in the injured joint rather than systemically. Early neutralization of IL-1 with intra-articular injection could reduce the cartilage degeneration and synovial inflammation after an articular fracture. In addition, the systemic injection of IL-1 receptor antagonist proved to benefit PTOA prevention [50]. Similar findings were reported in a previous study, wherein the systemic sgp130 (an antagonist of IL-6/serum IL-6 receptor complex) was found to have no effects on the prevention of cartilage degradation during antigen-induced arthritis in rats. The authors further suggested early neutralization of the IL-6/serum IL-6 receptor complex directly at the inflamed joint to prevent joint destruction [51]. That evidence indicates that intra-articular administration directly acting on the inflamed joint provides more efficacy in the prevention of joint destruction in PTOA.

Joint pain is the most common symptom of OA and is also the most frequent cause for illness-related doctor’s visits. Acute joint injury instantly stimulates the production of pro-inflammatory cytokines, which further predispose one to ECM degradation and pain. The joint pain in OA is typically aggravated by activity and usually contributes to physical disability [52]. In our study, EGCG-treated ACLT rats exhibited improved weight-bearing ability and increased running endurance compared to sham-operated rats, indicating an improvement in OA-related pain. In addition to alleviation inflammation, our results also indicated that intra-articular injection EGCG could decrease the arthritic symptoms after ACLT injury in the experimental PTOA model.

Chondrocyte death, including chondrocyte necrosis and apoptosis occurring soon after joint injury, is key to the pathogenesis of PTOA [5]. Chondrocyte necrosis takes place immediately after a chondral injury thanks to more than 15–20 MPa of stress and increases the necrotic rate in response to more stress [53]. Unlike chondrocyte necrosis, chondrocyte apoptosis usually occurs secondarily to the biochemical and biomechanical changes after ECM damage [54]. The damage to the cartilage matrix increases the stress to chondrocytes and triggers a sequence of catabolic reactions, including cytokine enhancement, inflammatory activation, and reactive oxygen species production [54]. The anti-inflammatory effect of EGCG that suppresses the production of pro-inflammatory cytokines, MMPs, and aggregates seems to be the main mechanism of prevention chondrocyte apoptosis. Besides the anti-catabolic effect, EGCG also has an anabolic effect on chondrocytes. Huang et al. indicated an anabolic effect of EGCG in an in vitro study using rabbit articular chondrocytes. They demonstrated that EGCG can effectively inhibit the hypertrophic differentiation of chondrocytes, and promote the growth and the synthesis of cartilage ECM by upregulating the gene expression of aggrecan, Col II, and SOX9 [55]. Jin et al. recently reported that EGCG and hyaluronic acid hybrid hydrogel synergistically promote chondrogenic regeneration by enhancing the expression of Col II, SOX9, and aggrecan, and reducing inflammatory gene expression, including IL-1β, MMP-13, and ADAMTS5, in 3D encapsulated porcine chondrocytes [56].

In addition to the anti-inflammation, we also observed that EGCG can also prevent chondrocyte apoptosis in OA by upregulating autophagy. Our data showed that intra-articular injection of EGCG inhibited the mTOR signal pathway and may activate autophagy function. Beclin1 and LC3, the major indicator for autophagosome formation, increased after EGCG treatment, also indicating an upregulation of autophagy. p62 is involved in the assembling and transport of ubiquitinated proteins and organelles into the lysosomes for degradation. Since p62 is mainly removed by autophagosomes, it is thought to be a negative indicator of autophagy [57]. We found that p62 expression was induced after OA injection but suppressed after ECGC treatment. The results of the TUNEL staining further showed that the chondrocyte apoptosis caused by ACLT was also significantly reduced after EGCG treatment. Based on the above, we suggested that the reduction of chondrocyte apoptosis mediated by activation of autophagy may be a potential mechanism of action of EGCG for OA prevention.

There was a limitation in our study. We just measured the autophagy-related parameters, but not the direct autophagy image. It is very hard to get direct autophagy images in histology. Therefore, we just measured the autophagy-related parameters.

## 5. Conclusions

In conclusion, our results showed that the intra-articular injection of EGCG reduced cartilage degradation in experimental OA animals. The activation of autophagy markers (mTOR, beclin-1, LC3, and p62), along with the reductions in chondrocyte apoptosis, and COX-2 and MMP-13 expression in the OA cartilage, were observed in the EGCG-treated knees. We demonstrated the EGCG could modulate the OA progression, reduce joint inflammation, and prevent chondrocyte apoptosis by promoting autophagy. Our findings further indicated that EGCG exerts a variety of biological properties that can prevent chondrocyte apoptosis and could be a viable therapeutic approach for PTOA.

## Figures and Tables

**Table 1:** The results of blood analysis.

	SGOT (U/L)	SGPT (U/L)	BUN (mg/dL)	CRE (mg/dL)	hsCRP (mg/L)
**Ctrl**	113 ± 16.21	62 ± 2.52	18.72 ± 0.77	0.45 ± 0.02	<0.012
**OA**	101 ± 18.29	59 ± 4.45	18.52 ± 1.05	0.40 ± 0.02	<0.012
**10 μM EGCG**	114 ± 16.36	64 ± 1.79	17.63 ± 0.83	0.42 ± 0.02	<0.012

**Table 2:** The results of OARSI. * *p* < 0.05 OA vs. control; ** *p* < 0.01 OA vs. control; # *p* < 0.05 OA vs. EGCG).

	Ctrl	OA	10 uM EGCG
Cartilage matrix loss (Structure)	0.80 ± 0.2	1.5 ± 0.3	0.8 ± 0.2
Cartilage degeneration score	0.2 ± 0.2	1.25 ± 0.6	0.6 ± 0.3
Proteoglycan content	1.2 ± 0.3	2.5 ± 0.3 **	1.4 ± 0.3 ^#^
Surface to Tidemark integrity	0.0	0.0	0.0
Additional features osteophyte	0.0	0.0	0.0
Mean	2.6 ± 0.2	5.25 ± 0.6 *	3.0 ± 0.29 ^#^

**Table 3:** Grading of chronic synovitis. * *p* < 0.05 OA vs. control; ** *p* < 0.01 OA vs. control; # *p* < 0.05 OA vs. EGCG.

L	Ctrl	OA	10 μM EGCG
Hyperplasia	0.25 ± 0.50	0.50 ± 0.55	0.50 ± 0.55
Inflammatory infiltration	0.00 ± 0.00	0.00 ± 0.00	0.00 ± 0.00
activation of synovial stroma	0.50± 0.58	0.50 ± 0.55	0.00 ± 0.00
**Total score**	**0.75 ± 0.50**	**1.00 ± 0.63**	**0.50 ± 0.55**
**R**	**Ctrl+DMSO**	**OA+DMSO**	**10 μM EGCG**
Hyperplasia	1.00 ± 0.00	1.33 ± 0.52	1.00 ± 0.00
Inflammatory infiltration	0.50 ± 0.58	0.83 ± 0.75	0.50 ± 0.55
activation of synovial stroma	1.00 ± 0.00	1.00 ± 0.00	1.00 ± 0.63
**Total score**	**2.50 ± 0.58**	3.17 ± 0.41 **	**2.50 ± 0.55 #**

## Data Availability

MDPI Research Data Policies.

## References

[1] Streich N.A., Zimmermann D., Bode G., Schmitt H. (2011). Reconstructive versus non-reconstructive treatment of anterior cruciate ligament insufficiency. A retrospective matched-pair long-term follow-up. Int. Orthop..

[2] Lohmander L.S., Englund P.M., Dahl L.L., Roos E.M. (2007). The long-term consequence of anterior cruciate ligament and meniscus injuries: Osteoarthritis. Am. J. Sports Med..

[3] Tawonsawatruk T., Sriwatananukulkit O., Himakhun W., Hemstapat W. (2018). Comparison of pain behaviour and osteoarthritis progression between anterior cruciate ligament transection and osteochondral injury in rat models. Bone Jt. Res..

[4] Hotham W.E., Malviya A. (2018). A systematic review of surgical methods to restore articular cartilage in the hip. Bone Jt. Res..

[5] Buckwalter J.A., Mankin H.J., Grodzinsky A.J. (2005). Articular cartilage and osteoarthritis. Instr. Course Lect..

[6] Zhang X., Bu Y., Zhu B., Zhao Q., Lv Z., Li B., Liu J. (2018). Global transcriptome analysis to identify critical genes involved in the pathology of osteoarthritis. Bone Jt. Res..

[7] D’Lima D., Hermida J., Hashimoto S., Colwell C., Lotz M. (2006). Caspase inhibitors reduce severity of cartilage lesions in experimental osteoarthritis. Arthritis Rheum..

[8] Huser C.A., Peacock M., Davies M.E. (2006). Inhibition of caspase-9 reduces chondrocyte apoptosis and proteoglycan loss following mechanical trauma. Osteoarthr. Cartil..

[9] Goodwin W., McCabe D., Sauter E., Reese E., Walter M., Buckwalter J.A., Martin J.A. (2010). Rotenone prevents impact-induced chondrocyte death. J. Orthop. Res..

[10] Li H., Yang H.H., Sun Z.G., Tang H.B., Min J.K. (2019). Whole-transcriptome sequencing of knee joint cartilage from osteoarthritis patients. Bone Jt. Res..

[11] Luk H.Y., Appell C., Chyu M.C., Chen C.H., Wang C.Y., Yang R.S., Shen C.L. (2020). Impacts of Green Tea on Joint and Skeletal Muscle Health: Prospects of Translational Nutrition. Antioxidants.

[12] Huang H.T., Cheng T.L., Lin S.Y., Ho C.J., Chyu J.Y., Yang R.S., Chen C.H., Shen C.L. (2020). Osteoprotective Roles of Green Tea Catechins. Antioxidants.

[13] Singh R., Ahmed S., Islam N., Goldberg V.M., Haqqi T.M. (2002). Epigallocatechin-3-gallate inhibits interleukin-1beta-induced expression of nitric oxide synthase and production of nitric oxide in human chondrocytes: Suppression of nuclear factor kappaB activation by degradation of the inhibitor of nuclear factor kappaB. Arthritis Rheum..

[14] Ahmed S., Rahman A., Hasnain A., Lalonde M., Goldberg V.M., Haqqi T.M. (2002). Green tea polyphenol epigallocatechin-3-gallate inhibits the IL-1 beta-induced activity and expression of cyclooxygenase-2 and nitric oxide synthase-2 in human chondrocytes. Free Radic Biol. Med..

[15] Rasheed Z., Anbazhagan A.N., Akhtar N., Ramamurthy S., Voss F.R., Haqqi T.M. (2009). Green tea polyphenol epigallocatechin-3-gallate inhibits advanced glycation end product-induced expression of tumor necrosis factor-alpha and matrix metalloproteinase-13 in human chondrocytes. Arthritis Res. Ther..

[16] Ahmed S., Wang N., Lalonde M., Goldberg V.M., Haqqi T.M. (2004). Green tea polyphenol epigallocatechin-3-gallate (EGCG) differentially inhibits interleukin-1 beta-induced expression of matrix metalloproteinase-1 and -13 in human chondrocytes. J. Pharmacol. Exp. Ther..

[17] Vankemmelbeke M.N., Jones G.C., Fowles C., Ilic M.Z., Handley C.J., Day A.J., Knight C.G., Mort J.S., Buttle D.J. (2003). Selective inhibition of ADAMTS-1, -4 and -5 by catechin gallate esters. Eur. J. Biochem..

[18] Marino G., Niso-Santano M., Baehrecke E.H., Kroemer G. (2014). Self-consumption: The interplay of autophagy and apoptosis. Nat. Rev. Mol. Cell Biol..

[19] Ren J., Zhang Y. (2018). Targeting Autophagy in Aging and Aging-Related Cardiovascular Diseases. Trends Pharmacol. Sci..

[20] Carames B., Taniguchi N., Otsuki S., Blanco F.J., Lotz M. (2010). Autophagy is a protective mechanism in normal cartilage, and its aging-related loss is linked with cell death and osteoarthritis. Arthritis Rheum..

[21] Caramés B., Taniguchi N., Seino D., Blanco F.J., D’Lima D., Lotz M. (2012). Mechanical injury suppresses autophagy regulators and pharmacologic activation of autophagy results in chondroprotection. Arthritis Rheum..

[22] Chen C.H., Ho M.L., Chang L.H., Kang L., Lin Y.S., Lin S.Y., Wu S.C., Chang J.K. (2018). Parathyroid hormone-(1-34) ameliorated knee osteoarthritis in rats via autophagy. J. Appl Physiol..

[23] Chou H.C., Chen C.H., Chou L.Y., Cheng T.L., Kang L., Chuang S.C., Lin Y.S., Ho M.L., Wang Y.H., Lin S.Y. (2020). Discoidin Domain Receptors 1 Inhibition Alleviates Osteoarthritis via Enhancing Autophagy. Int. J. Mol. Sci..

[24] Chen C.H., Ho M.L., Chang J.K., Hung S.H., Wang G.J. (2005). Green tea catechin enhances osteogenesis in a bone marrow mesenchymal stem cell line. Osteoporos Int..

[25] Lin R., Chen C., Wang Y., Ho M., Hung S., Chen I., Wang G. (2009). (-)-Epigallocatechin gallate inhibition of osteoclastic differentiation via NF-kappaB. Biochem. Biophys. Res. Commun..

[26] Chen C.H., Kang L., Lin R.W., Fu Y.C., Lin Y.S., Chang J.K., Chen H.T., Chen C.H., Lin S.Y., Wang G.J. (2013). (-)-Epigallocatechin-3-gallate improves bone microarchitecture in ovariectomized rats. Menopause.

[27] Lin S.Y., Kang L., Wang C.Z., Huang H.H., Cheng T.L., Huang H.T., Lee M.J., Lin Y.S., Ho M.L., Wang G.J. (2018). (-)-Epigallocatechin-3-Gallate (EGCG) Enhances Osteogenic Differentiation of Human Bone Marrow Mesenchymal Stem Cells. Molecules.

[28] Chen S.T., Kang L., Wang C.Z., Huang P.J., Huang H.T., Lin S.Y., Chou S.H., Lu C.C., Shen P.C., Lin Y.S. (2019). (-)-Epigallocatechin-3-Gallate Decreases Osteoclastogenesis via Modulation of RANKL and Osteoprotegrin. Molecules.

[29] Lin S.Y., Kang L., Chen J.C., Wang C.Z., Huang H.H., Lee M.J., Cheng T.L., Chang C.F., Lin Y.S., Chen C.H. (2019). (-)-Epigallocatechin-3-gallate (EGCG) enhances healing of femoral bone defect. Phytomedicine.

[30] Lin S.Y., Kan J.Y., Lu C.C., Huang H.H., Cheng T.L., Huang H.T., Ho C.J., Lee T.C., Chuang S.C., Lin Y.S. (2020). Green Tea Catechin (-)-Epigallocatechin-3-Gallate (EGCG) Facilitates Fracture Healing. Biomolecules.

[31] Chen C.H., Huang T.H., Cheng T.L., Chang C.F., Wang C.Z., Wu M.H., Kang L. (2017). Exercise training ameliorates glucosamine-induced insulin resistance in ovariectomized rats. Menopause.

[32] Chang J.K., Chang L.H., Hung S.H., Wu S.C., Lee H.Y., Lin Y.S., Chen C.H., Fu Y.C., Wang G.J., Ho M.L. (2009). Parathyroid hormone 1-34 inhibits terminal differentiation of human articular chondrocytes and osteoarthritis progression in rats. Arthritis Rheum..

[33] Gerwin N., Bendele A.M., Glasson S., Carlson C.S. (2010). The OARSI histopathology initiative—Recommendations for histological assessments of osteoarthritis in the rat. Osteoarthr. Cartil..

[34] Krenn V., Morawietz L., Häupl T., Neidel J., Petersen I., König A. (2002). Grading of chronic synovitis--a histopathological grading system for molecular and diagnostic pathology. Pathol. Res. Pract..

[35] Chen C.H., Kuo S.M., Tien Y.C., Shen P.C., Kuo Y.W., Huang H.H. (2020). Steady Augmentation of Anti-Osteoarthritic Actions of Rapamycin by Liposome-Encapsulation in Collaboration with Low-Intensity Pulsed Ultrasound. Int. J. Nanomed..

[36] Chou L.Y., Chen C.H., Lin Y.H., Chuang S.C., Chou H.C., Lin S.Y., Fu Y.C., Chang J.K., Ho M.L., Wang C.Z. (2020). Discoidin domain receptor 1 regulates endochondral ossification through terminal differentiation of chondrocytes. FASEB J..

[37] Kramer W.C., Hendricks K.J., Wang J. (2011). Pathogenetic mechanisms of posttraumatic osteoarthritis: Opportunities for early intervention. Int. J. Clin. Exp. Med..

[38] Tang Z., Yang L., Wang Y., Xue R., Zhang J., Huang W., Chen P.C., Sung K.L. (2009). Contributions of different intraarticular tissues to the acute phase elevation of synovial fluid MMP-2 following rat ACL rupture. J. Orthop. Res..

[39] Marks P.H., Donaldson M.L. (2005). Inflammatory cytokine profiles associated with chondral damage in the anterior cruciate ligament-deficient knee. Arthroscopy.

[40] Wojdasiewicz P., Poniatowski Ł.A., Szukiewicz D. (2014). The role of inflammatory and anti-inflammatory cytokines in the pathogenesis of osteoarthritis. Mediat. Inflamm..

[41] Ohishi T., Goto S., Monira P., Isemura M., Nakamura Y. (2016). Anti-inflammatory Action of Green Tea. Antiinflamm. Antiallergy Agents Med. Chem..

[42] Heinecke L.F., Grzanna M.W., Au A.Y., Mochal C.A., Rashmir-Raven A., Frondoza C.G. (2010). Inhibition of cyclooxygenase-2 expression and prostaglandin E2 production in chondrocytes by avocado soybean unsaponifiables and epigallocatechin gallate. Osteoarthr. Cartil..

[43] Leong D.J., Choudhury M., Hanstein R., Hirsh D.M., Kim S.J., Majeska R.J., Schaffler M.B., Hardin J.A., Spray D.C., Goldring M.B. (2014). Green tea polyphenol treatment is chondroprotective, anti-inflammatory and palliative in a mouse post-traumatic osteoarthritis model. Arthritis Res. Ther..

[44] Meki A., Sobhi A.B., Oraini M., Mehana E.-S., Deeb E. (2007). The Protective Effect of Green Tea Extract Against The Oxidative Stress of The Experimental Arthritic Rats. Damascus Univ. J Med. Sci..

[45] Bondeson J., Blom A.B., Wainwright S., Hughes C., Caterson B., Van den Berg W.B. (2010). The role of synovial macrophages and macrophage-produced mediators in driving inflammatory and destructive responses in osteoarthritis. Arthritis Rheum..

[46] Yun H.J., Yoo W.H., Han M.K., Lee Y.R., Kim J.S., Lee S.I. (2008). Epigallocatechin-3-gallate suppresses TNF-alpha -induced production of MMP-1 and -3 in rheumatoid arthritis synovial fibroblasts. Rheumatol. Int..

[47] Ahmed S., Pakozdi A., Koch A.E. (2006). Regulation of interleukin-1beta-induced chemokine production and matrix metalloproteinase 2 activation by epigallocatechin-3-gallate in rheumatoid arthritis synovial fibroblasts. Arthritis Rheum..

[48] Dekant W., Fujii K., Shibata E., Morita O., Shimotoyodome A. (2017). Safety assessment of green tea based beverages and dried green tea extracts as nutritional supplements. Toxicol. Lett..

[49] Furtado R.N., Oliveira L.M., Natour J. (2005). Polyarticular corticosteroid injection versus systemic administration in treatment of rheumatoid arthritis patients: A randomized controlled study. J. Rheumatol..

[50] Furman B.D., Mangiapani D.S., Zeitler E., Bailey K.N., Horne P.H., Huebner J.L., Kraus V.B., Guilak F., Olson S.A. (2014). Targeting pro-inflammatory cytokines following joint injury: Acute intra-articular inhibition of interleukin-1 following knee injury prevents post-traumatic arthritis. Arthritis Res. Ther..

[51] Boettger M.K., Leuchtweis J., Kümmel D., Gajda M., Bräuer R., Schaible H.G. (2010). Differential effects of locally and systemically administered soluble glycoprotein 130 on pain and inflammation in experimental arthritis. Arthritis Res. Ther..

[52] Lieberthal J., Sambamurthy N., Scanzello C.R. (2015). Inflammation in joint injury and post-traumatic osteoarthritis. Osteoarthr. Cartil..

[53] Milentijevic D., Rubel I.F., Liew A.S., Helfet D.L., Torzilli P.A. (2005). An in vivo rabbit model for cartilage trauma: A preliminary study of the influence of impact stress magnitude on chondrocyte death and matrix damage. J. Orthop. Trauma.

[54] Thomas C.M., Fuller C.J., Whittles C.E., Sharif M. (2007). Chondrocyte death by apoptosis is associated with cartilage matrix degradation. Osteoarthr. Cartil..

[55] Huang H., Liu Q., Liu L., Wu H., Zheng L. (2015). Effect of epigallocatechin-3-gallate on proliferation and phenotype maintenance in rabbit articular chondrocytes in vitro. Exp. Ther. Med..

[56] Jin Y., Koh R.H., Kim S.H., Kim K.M., Park G.K., Hwang N.S. (2020). Injectable anti-inflammatory hyaluronic acid hydrogel for osteoarthritic cartilage repair. Mater. Sci. Eng. C Mater. Biol. Appl..

[57] Lippai M., Lőw P. (2014). The role of the selective adaptor p62 and ubiquitin-like proteins in autophagy. Biomed. Res. Int..

